# Improved Extraction Methods to Isolate High Molecular Weight DNA From Magnaporthaceae and Other Grass Root Fungi for Long-Read Whole Genome Sequencing

**DOI:** 10.21769/BioProtoc.5245

**Published:** 2025-03-20

**Authors:** Michelle J. Grey, Jackie Freeman, Jason Rudd, Naomi Irish, Gail Canning, Tania Chancellor, Javier Palma-Guerrero, Rowena Hill, Neil Hall, Kim E. Hammond-Kosack, Mark McMullan

**Affiliations:** 1 Department of Organisms and Ecosystems, Earlham Institute, Norwich, England; 2 Rothamsted Research, Strategic Areas: Protecting Crops and the Environment, Plant Sciences for the Bioeconomy, West Common, Harpenden, UK; 3 Crop Science Centre, Department of Plant Sciences, University of Cambridge, Cambridge, UK; 4 Research Institute of Organic Agriculture FiBL, Frick, Switzerland

**Keywords:** Fungi, Magnaporthaceae, HMW DNA extraction, Long-read sequencing, Whole genome

## Abstract

This manuscript details two modified protocols for the isolation of long-stranded or high molecular weight (HMW) DNA from Magnaporthaceae (Ascomycota) fungal mycelium intended for whole genome sequencing. The Cytiva Nucleon PhytoPure and the Macherey-Nagel NucleoBond HMW DNA kits were selected because the former requires lower amounts of starting material and the latter utilizes gentler methods to maximize DNA length, albeit at a higher requirement for input material. The Cytiva Nucleon PhytoPure kit successfully recovered HMW DNA for half of our fungal species by increasing the amount of RNase A treatment and adding in a proteinase K treatment. To reduce the impact of pigmentation development, which occurs toward later stages of culturing, extractions were run in quadruplicate to increase overall DNA concentration. We also adapted the Macherey-Nagel NucleoBond HMW DNA kit for high-quality HMW DNA by grinding the sample to a fine powder, overnight lysis, and splitting the sample before washing the precipitated DNA. For both kits, precipitated DNA was spooled out pre-washing, ensuring a higher percentage of high-integrity long strands. The Macherey-Nagel protocol offers advantages over the first through the utilization of gravity columns that provide gentler treatment, yielding >50% of high-purity DNA strands exceeding 40 kbp. The limitation of this method is the requirement for a large quantity of starting material (1 g). By triaging samples based on the rate of growth relative to the accumulation of secondary metabolites, our methodologies hold promise for yielding reliable and high-quality HMW DNA from a variety of fungal samples, improving sequencing outcomes.

Key features

• Modified protocols for the extraction of high molecular weight fungal DNA suitable for long-read whole genome sequencing.

• Approximately 4 h to complete four samples in parallel (excluding lysis time).

• Optimized for the mycelium of Magnaporthaceae fungi, harvested before melanin (or secondary metabolite) buildup, but readily adaptable for other ascomycetes.

## Graphical overview


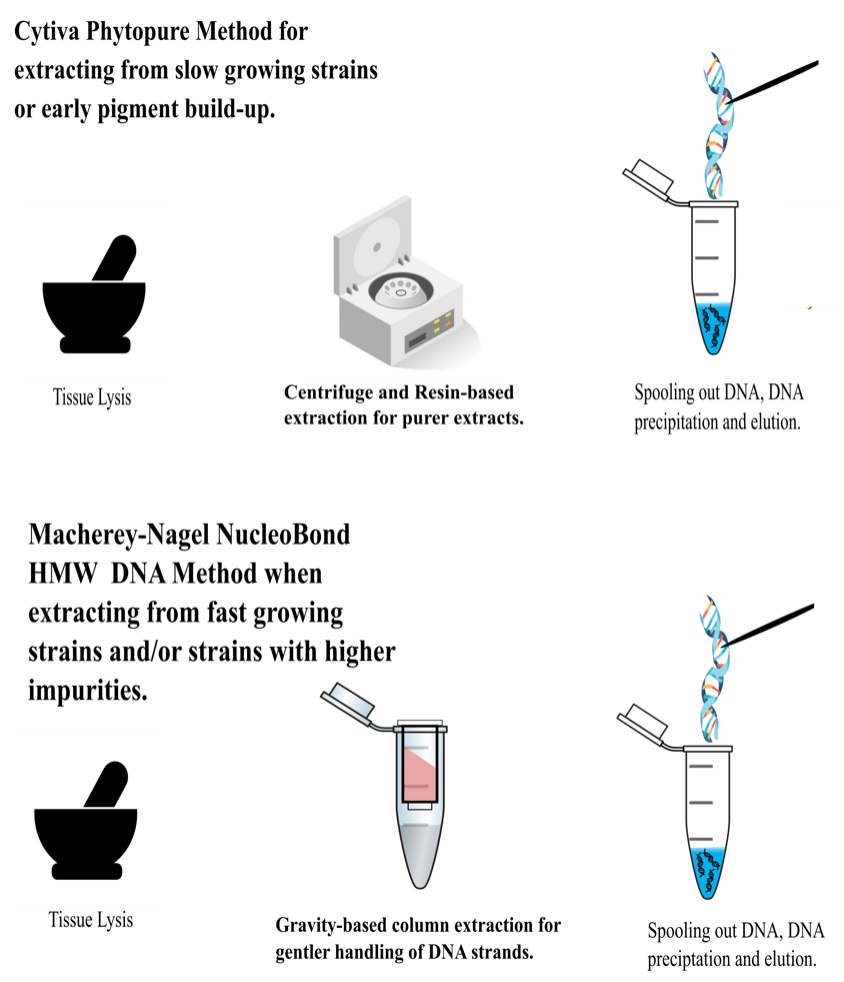


**Extraction process**

## Background

High-quality fungal genome assemblies play a crucial role in understanding the evolution of virulence and identifying genetic factors associated with pathogenic and non-pathogenic lifestyles [1–3]. Short read data tends to result in genome assemblies that are more fragmented because reads are unable to span structural variants and repetitive regions [4]. Fungi may be particularly impacted by structural genomic variation and giant mobile elements, which may be underestimated by current assemblies [5–6]. As such, demand has emerged for the generation of long-read data better able to span repetitive regions of the genome to improve assembly contiguity and the incorporation of such repetitive content. Producing long-read sequencing data is dependent on high molecular weight (HMW) DNA. In fungi, extracting the high concentrations of DNA needed whilst also maintaining strand length and integrity is challenging, perhaps due to their cell walls, production of secondary metabolites, or the often small amounts of starting material [7]. Several kits and methods were explored before settling on modified methods using the Cytiva Nucleon PhytoPure and the Macherey-Nagel HMW DNA kits (see Table S1). Our aim was to extract HMW DNA for PacBio sequencing in a volume higher than 13 μg. Harvesting the mycelium before pigmentation caused by the buildup of melanin and other secondary metabolites improves DNA quality after extraction using both modified methods. We have developed and optimized HMW DNA extraction methodologies for Magnaporthaceae species and other grass root fungal species.

**Part I**

**Cytiva Nucleon PhytoPure kit modified protocol**

## Materials and reagents

**Reagents**

1. Cytiva Nucleon PhytoPure, pack of 50 × 1.0 g (Cytiva, catalog number: RPN8511); this kit contains Reagent 1, Reagent 2, and Phytopure resin

2. Propanol-2-OL (Merck Life Sciences, catalog number: 33539-2.5LM, CAS 67-63-0)

3. Chloroform, analytical grade (Merck Life Sciences, catalog number: 32211-2.5LM, CAS 67-66-3)

4. Ethanol, denatured (VWR, catalog number: 01000940, CAS 64-17-5)

5. Invitrogen PureLink RNase A, 20 mg/mL (Fisher Scientific, catalog number: 10618703)

6. Proteinase K, 30 mg/mL (Thermo Scientific, catalog number: 11501515)

7. Low-TE elution buffer, 1× solution, pH 8.0 (Fisher Scientific, catalog number: 10647633)

8. Potato dextrose broth (Formedium, catalog number: PDB0102)

10. Qubit high sensitivity assay (Invitrogen, catalog number: Q23854)

11. Qubit broad range assay (Invitrogen, catalog number: Q32853)

**Solutions**

1. 70% ethanol solution (EtOH) (see Recipes)

**Recipes**

**1. 70% EtOH**

**Table:** 

Reagent	Final concentration	Quantity or Volume
Ethanol, 99.9% pure	n/a	30 mL
Sterilized purified water	n/a	20 mL
Total (optional)	n/a	50 mL

*Note: This solution should be made up fresh on the day of the extraction.*

**Laboratory supplies**

1. 2 mL DNA LoBind Eppendorf tubes (Fisher Scientific, catalog number: 10031282)

2. Filter p1000 tips (Elkay Laboratory Products, catalog number: AER-2REF-S96)

3. Filter p200 tips (Elkay Laboratory Products, catalog number: AER-REF-S96)

4. Paper tissue medical wipes (Bunzle Cleaning and Hygiene Supplies, catalog number: 066080)

5. Disposable PES bottle top filter 0.2 μm, 500 mL capacity (Fisher Brand, catalog number: 15983307)

6. 0.22 μm bottle top filter (Fisherbrand disposable PES) (Fisher Scientific, catalog number: 15993307)

## Equipment

1. Thermomixer-mixer HC (Starlab Smart Instruments, model: S8012-0000)

2. 100–1,000 mL ErgoOne pipette (Starlabs, model: S7100-1000)

3. 20–200 mL ErgoOne pipette (Starlabs, model: S7100-2200)

4. Centrifuge (Eppendorf, model: 54520000060 mini spin plus)

5. 250 mL glazed mortar and pestle (Haldenwanger, model: 55/3/glazed)

6. Freezer -20 °C and freezer -80 °C

7. Vortex mixer (Stuart Science Equipment, model: SA8)

8. Vacuum pump (WooSung, model: W2v20)

9. Growth cabinet (Binder, model: BD400)

10. Orbital shaker (SciQuip, model: SP2250-07)

11. NanoDrop One Microvolume UV-Vis spectrophotometer (Thermo Scientific, model: NC-ONEC-W)

12. Qubit fluorometer (Invitrogen, model: Qubit 3.0, catalog number: Q32854)

13. Autoclave (Astell, model: ASB30019293)

14. Orbital Shaker (SciQuip model: shaker 07)

15. Growth cabinet (Binder, model: BD400)

16. Vacuum pump (JungWoo, model: Woo Sung Automa 20)

17. 500 mL borosilicate glass Erlenmeyer flasks (Fisher Scientific, catalog number: 15429113)

18. Femto Pulse system (Agilent, catalog number: M5330AA)

## Procedure

**A1. Biological materials**

1. In order to obtain the fungal mycelial mass required for DNA extraction, place four mycelial plugs (6 mm in diameter) in 500 mL of potato dextrose broth (PDB) and grow for 7–14 days shaking at 140 rpm on an orbital shaker in a growth cabinet protected from light, at 22 °C.

2. To reach the required gDNA concentration, up to 1 g of flash-frozen mycelium is required. When enough mycelium for the extraction plus backup material has grown, harvest ~3 g of mycelium using a 0.22 μm bottle top filter and vacuum pump.

3. Rinse the mycelium with sterilized purified water to remove any residue of PDB.

4. Cut agar plugs from the harvested mycelium and place them in an autoclave bag. Flash freeze the harvested material in liquid nitrogen and store in a -80 °C freezer until ready to process.

**A2. Before starting**

1. Thaw the required amount of RNase A and Proteinase K on ice, if kept frozen.

2. Pre-cool freshly aliquoted propanol-2-OL in freezer at -20 °C.

3. Pre-cool freshly aliquoted chloroform in freezer at -20 °C.

4. Make up a fresh batch of 70% EtOH.

5. Ensure mortars and pestles have been cleaned and sterilized in an autoclave at 121 °C for 15 min.

6. Collect dry ice and keep in a polystyrene box in the fume hood.

7. Preheat reagent 1 to 50 °C.

**B. Sample preparation**

1. Working over dry ice in a fume hood, grind 100 mg of flash-frozen mycelium with a sterilized mortar and pestle until a fine powder is formed. This powder should resemble talcum powder in consistency. Transfer this to a labeled 2 mL Eppendorf tube.

*Note: If working with low-yielding strains, run samples in quadruplicate and pool the extracts together when completed. This would require 400 mg of starting material to be ground.*

**C. Cell lysis**

1. Add 600 μL of preheated Reagent 1 and 20 μL of RNase A to the sample in the 2 mL tube. Mix well with a vortex mixer or by inverting the tube 5–10 times, until the mycelium is homogenized with the buffers and enzymes. Ensure all the mycelium has been incorporated into the buffer. Incubate at 50 °C for 30 min, mixing at 450 rpm using the HC mixer.

*Note: For low-yielding strains, the lysis time can be extended overnight without harm to the DNA. Strains have been incubated for up to 12 h to improve yield.*

2. Once lysis incubation is completed, add 200 mL of Reagent 2 and mix by gentle inversion 5–10 times until a consistent solution is acquired.

3. Add 10 μL of proteinase K (30 mg/mL = 600 U/mL) and incubate at 65 °C for 1 h with shaking at 450 rpm.

**D. DNA precipitation**

1. Place samples on ice for 20 min.

2. While working in a fume hood, add 500 μL of pre-cooled chloroform and 100 μL of Cytiva Nucleon PhytoPure DNA extraction resin suspension. Mix by gentle rotation in a rotating mixer for 10 min at room temperature.

3. Centrifuge in a benchtop microfuge at 1,300× *g* for 10 min. Transfer the upper aqueous phase to a new, labeled 2 mL tube. Take care not to disturb the interface. Be conservative and leave some of the upper phase behind rather than carrying over chloroform or resin (expect ~500 μL).

4. Add 1× volume of pre-cooled propanol-2-OL to each tube. Mix the tube by inversion 5–10 times and incubate at -20 °C for 15 min.

5. Centrifuge in a benchtop microfuge at 4,000× *g* for 10 min. Discard the supernatant without disturbing the pellet. Leave the cap open on the tube and set it upright in a tube rack to air dry the sample for 2 min.

**E. DNA wash**

1. Wash the pellet with 1 mL of the prepared 70% EtOH. Invert the tube five times and centrifuge in a benchtop microfuge at 4,000× *g* for 20 min to collect the pellet.

2. Pour off the ethanol without disturbing the pellet.

3. Repeat the wash steps two more times for a total of three washes.

4. Dry the sample by leaving the caps open for 5 min or until you no longer see droplets of ethanol present in the tube or around the pellet. Make sure to check the inside of the caps where ethanol can pool. Use a sterile medical wipe to carefully remove large droplets of ethanol on the caps or near the top of the tube. Do not put the medical wipe near the DNA pellet.

5. Resuspend the pellet in 100 μL of elution buffer. In our experience, it is better to let the pellet dissolve into the buffer by leaving it overnight in a refrigerator. Quantify the following day.

**Part II**

**Macherey-Nagel NucleoBond HMW DNA kit modified protocol**

## Materials and reagents

**Reagents**

1. Macherey-Nagel NucleoBond HMW DNA, high molecular weight DNA from diverse sample materials (Macherey-Nagel, catalog number: 740160.20); this kit contains buffers H1–H5, HE elution buffer, RNase A, and Proteinase K

2. Propanol-2-OL (Merck Life Sciences, catalog number: 33539-2.5LM, CAS 67-63-0)

3. Ethanol, denatured (VWR, catalog number: 01000940, CAS 64-17-5)

4. Sterilized, purified water

5. Potato dextrose broth (Formedium, catalog number: PDB0102)

9. Liquid nitrogen (CAS 7727379)

10. Qubit high sensitivity assay (Invitrogen, catalog number: Q23854)

11. Qubit broad range assay (Invitrogen, catalog number: Q32853)

**Solutions**

1. 70% ethanol solution (EtOH) (see Recipes)

**Recipes**

**1. 70% EtOH**

**Table:** 

Reagent	Final concentration	Quantity or Volume
Ethanol, 99.9% pure	n/a	30 mL
Sterilized purified water	n/a	20 mL
Total (optional)	n/a	50 mL

*Note: This solution should be made up fresh on the day of the extraction.*

**Laboratory supplies**

1. 50 mL Falcon tubes (Corning, catalog number: 430790)

2. 2 mL DNA LoBind Eppendorf tubes (Fisher Scientific, catalog number: 10031282)

3. Filter p1000 tips (Elkay Laboratory Products, catalog number: AER-2REF-S96)

4. Filter p200 tips (Elkay Laboratory Products, catalog number: AER-REF-S96)

5. 10 mL disposable pipette (Corning, catalog number: 4488)

6. 5 mL disposable pipette (Corning, catalog number: 4487)

7. Paper tissue medical wipes (Bunzle Cleaning and Hygiene Supplies, catalog number: 066080)

8. 0.22 μm bottle top filter (disposable PES) (Fisherbrand, Fisher Scientific, catalog number: 15993307)

## Equipment

1. Thermomixer-mixer HC (Starlab Smart Instruments, model: S8012-0000)

2. 250 mL glazed mortar and pestle (Haldenwanger, model: 55/3/glazed)

3. 100–1,000 mL ErgoOne pipette (Starlabs, model: S7100-1000)

4. 20–200 mL ErgoOne pipette (Starlabs, model: S7100-2200)

5. FAST pipette controller (Starlabs, model: S7166-0010)

6. Centrifuge (Eppendorf, model: 54520000060)

7. Freezers able to maintain -20 °C and -80 °C

8. Vortex mixer (Stuart Science Equipment, model: SA8)

9. NanoDrop One Microvolume UV-Vis spectrophotometer (Thermo Scientific, model: ND-ONEC-W)

10. Qubit fluorometer (Invitrogen, model: Qubit 3.0, catalog number: Q32854)

11. Autoclave (Astell model: ASB30019293)

12. 500 mL borosilicate glass Erlenmeyer flasks (Fisher Scientific, catalog number: 15429113)

13. Femto Pulse system (Agilent, model: M5330AA)

14. Orbital shaker (SciQuip, model: shaker 07)

15. Growth cabinet (Binder, model: BD400)

16. Vacuum pump (JungWoo, model: Woo Sung Automa 20)

## Procedure

**A1. Prepare biological material as described in Method 1, step A1.**

**A2. Before starting**

1. Preheat the buffer H1 to 55 °C.

2. Heat the HC mixer to 55 °C.

3. Pre-chill sterilized mortar, pestle, measuring spoon, and a labeled 50 mL Falcon tube in a polystyrene box containing dry ice. Place this in the fume hood.

4. Prepare 70% EtOH

**B. Sample preparation**

1. Working over dry ice in a fume hood, grind 1 g of flash-frozen mycelium using a sterilized mortar and pestle until a fine powder is formed. This powder should resemble talcum powder in consistenc*y*. Transfer this to a labeled 50 mL Falcon tube and keep on dry ice until ready to start lysis.

**C. Cell lysis**

1. Add 2.5 mL of preheated lysis buffer H1 and 100 μL of proteinase K to each tube. If more sample material needs to be processed (e.g., with low-yielding samples), increase volumes proportionally in this step and when adjusting the binding conditions.

2. Vortex tubes for 5 s. Make sure that large amounts of the sample do not stick to the inner wall of the centrifuge tube.

3. Incubate at 50 °C for 30 min, shaking at 450 rpm using the HC mixer.

*Note: For low-yielding strains, the lysis time can be extended overnight without harm to the DNA. Strains have been incubated for up to 12 h to improve yield.*

**D. RNA digestion**

1. Once lysis incubation is completed, add 50 μL of RNase A to each tube and mix by inversion until a consistent solution is acquired.

2. Incubate for 5 min at room temperature.

**E. Column steps**

1. For each sample, combine a Macherey-Nagel NucleoBond HMW column (including filter) with a plastic washer and arrange the combination on a labeled 50 mL Falcon tube. Set these in a stand. While setting this up, ensure that the plastic washer does not seal the waste vessel airtight as stated in the kit’s protocol.

2. Equilibrate the filters and columns by adding 12 mL of buffer H2 to the upper rim of the column filters and make sure the complete filter and the silica matrix are pre-wet.

3. Let gravity perform these steps while using these columns. Do not use a vacuum.

4. Discard the flowthrough.

5. Add 10 mL of buffer H2 to each sample and mix by inverting the tube gently 5 times. (If you added more starting material at the start of this protocol, this is where you will need to increase volume proportionally.)

6. Load samples, trying to avoid any debris into the center of the column filters, and let the lysate pass the silica matrix by gravity flow.

7. Discard the flowthrough.

8. Add 6 mL of buffer H3 to the rim of the column filters and let the buffer pass the filter and the silica.

9. Discard the flowthrough and the column filter once the buffer has passed the silica.

10. Wash the column without filter with 12 mL of buffer H4.

11. Discard the flowthrough.

12. Place the HMW column on top of a clean and labeled 50 mL Falcon tube.

13. Elute DNA with 5 mL of buffer H5.

**F. Precipitate DNA**

1. Using a wide-bore 1,000 μL pipette tip, divide the DNA solution into 5 × 2 mL tubes (1,000 μL/tube). Use caution when pipetting to keep from shearing your long strands of DNA.

2. Add 1,000 μL of chilled propanol-2-OL to each tube.

3. Mix by carefully and slowly inverting the tube 10 times.

4. Using a sterilized glass rod, spool out visible DNA strands into a labeled tube with 50 μL of Low TE or HE elution buffer. There is no need to wash spooled DNA strands. Set this into the fridge when finished. (This is the final tube into which you will pool the washed DNA once completing steps K5, K6, L1–L7, and M1–M3.

5. Centrifuge what remains in the tubes after twisting out noticeable DNA at 5,000× *g* for 10 min at room temperature.

6. Carefully discard the supernatant. Pellets will be difficult to see.

**G. Wash DNA pellet**

1. Add 500 μL of 70% EtOH solution to each DNA pellet.

2. Centrifuge at 5,000× *g* for 5 min at room temperature.

3. Carefully discard the supernatant completely.

4. Add 25 μL of 70% EtOH to each DNA pellet for a second wash.

5. Centrifuge at 7,000× *g* for 5 min at room temperature.

6. Carefully discard the supernatant completely.

7. Place tubes with the lids open upright in a tray to dry the pellet by incubating at room temperature until the ethanol has evaporated (~5 min). Check the inside of the lids of the tubes for excess droplets. Using sterilized medical tissue, dab any ethanol droplets. Do not overdry the pellet, as this can lead to degradation of the DNA and difficulties in resuspension.

**H. Resuspend DNA**

1. Add ≤20 μL of low TE or HE elution buffer to the DNA pellets in each tube and carefully resuspend the DNA by gently swirling the tube. Make sure the pellet is immersed in the buffer.

*Note: Remember you have 50 μL of the spooled DNA from step K4 already in the fridge. This will give you a final volume of 150 μL.*

2. Leave DNA overnight in the refrigerator (4 °C) for complete resuspension. In the morning, combine the DNA into your final tube (to which you have already added the DNA strands previously).

3. Use wide-bore pipette tips for all further downstream applications when handling DNA.

**I. DNA assessment**

1. Use a NanoDrop to assess the purity of the DNA after letting the pellets rest in elution buffer overnight.

2. Check the amount of DNA using a Qubit HS (high sensitivity) or BR (broad range) assay.

3. Check the length and integrity of the strands using a Femto analyzer.

## Validation of protocol

This protocol has been used and validated in the following research article:

• Hill et al. [1]. Evolutionary genomics reveals variation in structure and genetic content implicated in virulence and lifestyle in the genus Gaeumannomyces.

DNA quantity and quality were assessed using three techniques for nucleic acid quantification: UV spectrophotometry using a NanoDrop spectrophotometer, dsDNA specific fluorimetry using a Qubit fluorometer, and DNA integrity and length of strands using a Femto Pulse parallel capillary electrophoresis system. DNA purity was assessed by reading A_260/230nm_ and A_260/280nm_ on the NanoDrop instrument (Table 1). When determining the purity of each extraction, it is important to acknowledge the difference between the Qubit DNA concentration readings and the Nanodrop. Differences greater than 5% indicate impurity in the DNA extracts. On average, the Cytiva Nucleon PhytoPure kit produces greater differences than the Macherey-Nagel NucleoBond HMW DNA kit (Table 1).

After assessing whether DNA concentration and purity pass minimum requirements, the relative success of each extraction for sequencing is then determined by assessing strand length, which impacts downstream library preparation (Table 1). Samples with a greater proportion of shorter strands (at a cutoff of 40 kbp) are constructed using the 10–15 kb library preparation, and those with a greater proportion of larger strands are constructed using the 15–20 kb library preparation kit. Strand length will impact genome assembly contiguity. However, given the strand lengths attained here and the downstream biological questions, low input DNA extraction and shorter fragment length libraries did not impact our ability to address questions on genome evolution [6] (see Table S2).

**Table 1.** DNA quantity and quality mean (SD) values obtained from the Cytiva Nucleon PhytoPure kit (n = 11 each with quadruple replicates per the sample, pooled together) and the Macherey-Nagel NucleoBond HMW kit (n = 10). Nanodrop Qubit difference is the amount of DNA (ng/μL) estimated from the Nanodrop subtracted from the estimated amount of DNA (ng/μL) from the Qubit reading. Large differences indicate contamination in the extract. Femto pulse results characterize the percentage of each sample made up of given strand lengths.

Extraction method (n)	Qubit DNA	Nanodrop Qubit difference	260/280 Nanodrop	260/230 Nanodrop	Total DNA	FEMTO strand length 0–39,999 bp	FEMTO strand length 40,000–400,000 bp	N50	Largest contig
Units	ng/μL	ng/μL	1	1	μg	%	%	Mbp	Mbp
CN PhytoPure^1^ count: 11	63.0 (33.9)	-523.8 (430.0)	1.9 (0.1)	1.1 (0.3)	9.8 (2.2)	59.4 (13.8)	32.3 (16.5)	6.3 (1.0)	10.4 (2.2)
M-N NucleoBond^2^ count: 10	100.7 (83.0)	-160.4 (174.0)	1.8 (0.1)	1.7 (0.6)	10.6 (12.1)	56.9 (23.2)	38.3 (21.3)	5.2 (1.9)	7.9 (2.3)
REM: Bracket values denote standard deviation of mean values.									

**^1^** Cytiva Nucleon PhytoPure © 2024 Cytiva

^2^NucleoBond DNA/RNA/Protein © Copyright 2021 by MACHEREY-NAGEL GmbH & Co. KG

Perhaps because of the amounts of starting material required, 50% of the samples extracted using the Cytiva Nucleon PhytoPure DNA were successfully constructed for PacBio HiFi sequencing with a starting input of 2–5.5 μg at 18–20 ng/μL and manually sheared to between 15 and 20 kb using the Megaruptor 3 instrument (Diagenode, P/N B06010003), compared to 70% of the Macherey-Nagel NucleoBond HMW DNA.

Notably, 90% of the Macherey-Nagel extracts met the optimal purity ratio (260/280), compared to approximately 30% of the Cytiva Nucleon PhytoPure extracts. Nevertheless, sequencing results showed that the Cytiva Nucleon PhytoPure extracts, prepared by shearing between 15 and 20 kb, yielded an average N50 of 6.6 Mbp. In contrast, the Macherey-Nagel extracts, prepared using the same shearing method, had an average N50 of 5.2 Mbp. Overall, Cytiva Nucleon PhytoPure method samples exhibited longer contigs, averaging 10.4 Mbp regardless of the shearing size, while the longest contigs for the Macherey-Nagel method samples averaged 7.9 Mbp.

In initial tests of the two methods on identical ascomycete strains, the modified Macherey-Nagel method attained a higher proportion of strand lengths greater than 40 kbp (see Table S1). This demonstrates a reduction in the level of degradation or shearing of strands using the Macherey-Nagel kit, which is most likely due to the difference in the mechanics of each kit. Using these methods on different strains and species of our project, we once saw a similar set of results with the Macherey-Nagel NucleoBond HMW DNA method averaging a higher percentage of strands >40 kbp and less degradation overall (Table 2). This could be due to sample quality and the larger amount of input sample for the Macherey-Nagel method. It is crucial to consider our triaging strategy in interpreting these results, as the Cytiva Nucleon PhytoPure kit requires significantly less starting material.

**Table 2.** Average strand lengths

Modified kit	Average % strands <39,999 bp	Average % strands >40,000 bp
CN PhytoPure*	59.4	32.3
M-N NucleoBond*	56.9	38.3

*Note: The sample size was 400 mg using the Cytiva Nucleon PhytoPure method and 1,000 mg using the Macherey-Nagel NucleoBond HMW method.*

## General notes and troubleshooting

**General notes**

1. Our protocol outlines two different methodologies for extracting HMW DNA from Magnaporthaceae and other grass root filamentous fungi. Initially, fungi were grown on solid media and subsequently transferred to liquid media for biomass expansion. Out of 20 strains, we successfully passed 10 through the stringent PacBio quality control using the modified Cytiva Nucleon PhytoPure method. The remaining 10 strains required the modified Macherey-Nagel NucleoBond HMW DNA method. Given sufficient starting material, we recommend the modified Macherey-Nagel NucleoBond HMW DNA method for consistent high-quality, long-stranded DNA. When working with limited amounts of starting material, we recommend using the modified Cytiva Nucleon PhytoPure method. We believe that following these protocols will yield good results for other ascomycetes.

The two methods differ in several aspects:

• Amount of starting material required.

• Consistency across strains.

• Time needed for growing/collecting sufficient mycelium.

• DNA integrity and purity

2. Sometimes, it is visually obvious after sitting overnight that the DNA is not pure, as it may appear gloopy, colored, and viscous. When our strains reacted to the extraction in this manner, we used the modified Macherey-Nagel NucleoBond HMW method.

3. Melanin development plays a critical role in the infectivity and pathogenicity of pathogenic fungi. The accumulation of this molecule is still being studied but it is known that it is a necessary component of host cell cuticle and epidermal cell wall penetration [8]. In *Magnaporthe oryzae*, melanin buildup assists with strengthening chitin walls. In *Gaeumannomyces tritici*, not only do fungal cellulolytic and pectinolytic enzymes aid in the infection of host cells but melanin is also required, particularly in hyphae, where the formation of melanized appressoria-like structures called hyphopodia occur [9–10]. As important as this is to pathogenic fungi, it is problematic when extracting high-quality DNA. We found it best to harvest the mycelium before the buildup of melanin can occur. This presents a time constraint on how much material bulking can occur. If melanin or the accumulation of other secondary metabolites make longer bulking periods impossible, use the Cytiva Nucleon PhytoPure modified protocol. Use the Macherey-Nagel NucleoBond HMW DNA modified protocol when growth times and secondary metabolite accumulation are not a concern, or when large-scale parallelization of culturing is feasible.

**Troubleshooting**

Problem 1: Viscous or gloopy extract.

Possible cause: Leftover polysaccharides in extraction.

Solution: Use the modified Macherey-Nagel NucleoBond HMW protocol.

Problem 2: Low yield of DNA.

Possible cause: Incomplete lysis.

Solution: Ensure you are grinding the material to a fine powder and leave the lysis to incubate overnight.

Problem 3: Degraded or sheared strands of DNA.

Possible cause: The mycelium was not kept cold when preparing for lysis or the sample was handled too aggressively after lysis.

Solution: Ensure you keep your mycelium frozen during the steps preceding the lysis. Use extreme care with pipetting post-lysis.

## Supplementary information

The following supporting information can be downloaded here:

1. Table S1. Alternative methodologies and commercial kits evaluated that failed to pass quality control

2. Table S2. Metrics used to evaluate each sample’s library construction method.
